# Two new species and one new record of *Perennicordyceps* (*Polycephalomycetaceae*, *Hypocreales*) from China

**DOI:** 10.3897/mycokeys.136.199287

**Published:** 2026-07-03

**Authors:** Yu Yang, Jing-Yi Zhang, Hong-Xiu Liu, Kevin D. Hyde, Yong-Zhong Lu, Yin-Ping Gong, Bang-Xi Zhang, Yuan-Pin Xiao

**Affiliations:** 1 School of Food and Pharmaceutical Engineering, Guizhou Institute of Technology, Guiyang 550025, China Innovative Institute for Plant Health / Key Laboratory of Green Prevention and Control on Fruits and Vegetables in South China, Ministry of Agriculture and Rural Affairs, Zhongkai University of Agriculture and Engineering Guangzhou China; 2 Guizhou Key Laboratory of Agricultural Microbiology, Guizhou Academy of Agricultural Sciences, Guiyang 550009, China Guizhou Key Laboratory of Agricultural Microbiology, Guizhou Academy of Agricultural Sciences Guiyang China; 3 Soil and Fertilizer Research Institute, Guizhou Academy of Agricultural Sciences, Guizhou, Guiyang 550006, China Soil and Fertilizer Research Institute, Guizhou Academy of Agricultural Sciences Guiyang China; 4 Innovative Institute for Plant Health / Key Laboratory of Green Prevention and Control on Fruits and Vegetables in South China, Ministry of Agriculture and Rural Affairs, Zhongkai University of Agriculture and Engineering, Guangzhou, 510225, China Center of Excellence in Fungal Research, Mae Fah Luang University Chiang Rai Thailand; 5 Center of Excellence in Fungal Research, Mae Fah Luang University, Chiang Rai, 57100, Thailand School of Biological Science and Food Engineering, Chuzhou University Chuzhou China; 6 Department of Biology, Faculty of Science, Chiang Mai University, Chiang Mai, 50200, Thailand Department of Biology, Faculty of Science, Chiang Mai University Chiang Mai Thailand; 7 School of Biological Science and Food Engineering, Chuzhou University, Chuzhou 239000, China School of Food and Pharmaceutical Engineering, Guizhou Institute of Technology Guiyang China

**Keywords:** Entomopathogenic fungi, morphology, multigene phylogeny, *
Perennicordyceps
*, *
Polycephalomycetaceae
*

## Abstract

*Perennicordyceps* is a genus of clavicipitoid fungi in *Polycephalomycetaceae*, comprising primarily entomopathogenic species, with some species also associated with fungal hosts. Its diversity and host range remain poorly understood, particularly in biodiversity-rich regions such as southwestern China. In this study, two new species of *Perennicordyceps* are described based on morphological characteristics and multigene phylogenetic analyses (nrLSU, ITS, nrSSU, *tef*-1α, *rpb*1, and *rpb*2). A new host record is also reported, expanding the known ecological range of the genus. Phylogenetic analyses place the new taxa within *Perennicordyceps* as distinct, well-supported lineages. Morphological comparisons with related species further support their recognition as novel taxa. This study improves the understanding of species diversity, host associations, and evolutionary relationships within *Polycephalomycetaceae*.

## Introduction

Clavicipitoid fungi represent an important lineage within *Hypocreales* (*Sordariomycetes*, *Ascomycota*), encompassing diverse taxa associated with insects, fungi, and plants (Humber 2008; Kepler et al. 2012a, 2012b; Quandt et al. 2014; Spatafora et al. 2015; Xiao et al. 2023). Traditionally treated within a broad concept of *Clavicipitaceae*, this group was later resolved into several distinct families, including *Cordycipitaceae*, *Ophiocordycipitaceae*, and *Clavicipitaceae*, based on morphological and molecular phylogenetic evidence (Sung et al. 2007a, 2007b; Kepler et al. 2013). More recent multigene analyses demonstrated that *Polycephalomyces*, *Perennicordyceps*, and *Pleurocordyceps* form a distinct lineage, now recognized as *Polycephalomycetaceae*, with *Polycephalomyces* representing the type genus of the family (Xiao et al. 2023). *Polycephalomyces* was established by Kobayasi (1941), with *Po. formosus* designated as the type species, and is characterized by polycephalous synnemata.

Within *Polycephalomycetaceae*, several genera, including *Dingleyomyces* and *Paradingleyomyces* and the recently established *Sorobiellomyces*, have been established in recent years, reflecting increasing taxonomic resolution and ecological diversity within the family, including taxa parasitic on both insects and other fungi (Johnston and Park 2023; Wang et al. 2024; Zhang et al. 2026b). In addition, *Torrubiellomyces*, originally placed in *Ophiocordycipitaceae* (Araújo et al. 2022), has been phylogenetically recovered as closely related to *Polycephalomycetaceae* in subsequent analyses, including the present study (Wei et al. 2026). Members of this family exhibit notable ecological diversity, occurring as entomopathogens as well as parasites of fungi, particularly in tropical and subtropical regions (Matočec et al. 2014; Wang et al. 2021; Xiao et al. 2023, 2024; Tang et al. 2025). Among these genera, *Perennicordyceps* represents a distinct lineage that has attracted increasing taxonomic and ecological attention (Matočec et al. 2014; Xiao et al. 2023).

The genus *Perennicordyceps* was established by Matočec et al. (2014) to accommodate four species formerly assigned to *Polycephalomyces*, namely *Pe. prolifica*, *Pe. cuboidea*, *Pe. Paracuboidea*, and *Pe. ryogamiensis* (Schoch et al. 2012; Matočec et al. 2014). Species of *Perennicordyceps* are primarily entomopathogenic, occurring on insect larvae or pupae, especially those of *Coleoptera* and *Lepidoptera* (Matočec et al. 2014; Xiao et al. 2023; Chen et al. 2024). They are characterized by stromatic ascomata with superficial perithecia and acremonium- or hirsutella-like asexual morphs and form a well-supported lineage within *Polycephalomycetaceae* based on multigene phylogenetic analyses (Matočec et al. 2014; Xiao et al. 2023).

Subsequent studies have expanded both the taxonomic diversity and known host range of *Perennicordyceps*. *Perennicordyceps lutea* was described from *Ophiocordyceps sinensis* in China, while *Pe. elaphomyceticola*, a fungicolous species associated with *Elaphomyces*, was recombined in *Perennicordyceps* based on morphological and phylogenetic evidence (Xiao et al. 2023). *Perennicordyceps zongqii*, collected from lepidopteran larvae in karst regions of China, further expanded the known insect-host range of the genus (Chen et al. 2024). More recently, *Cordyceps szemaoensis* was shown to be conspecific with *Pe. elaphomyceticola* and was recombined as *Pe. szemaoensis*, further clarifying species boundaries within the genus (Tang et al. 2025). Current records indicate that *Perennicordyceps* is associated with both insect and fungal hosts (Matočec et al. 2014; Xiao et al. 2023; Chen et al. 2024; Tang et al. 2025). Together, these studies indicate that *Perennicordyceps* possesses considerable ecological diversity, although its diversity and host associations still require further investigation.

During surveys of entomopathogenic fungi and associated fungi from China, five specimens morphologically resembling *Perennicordyceps* were collected from coleopteran larvae, lepidopteran cocoons, and *Ophiocordyceps* species. Phylogenetic analyses based on a combined dataset of nrLSU, ITS, nrSSU, *tef*-1α, *rpb*1, and *rpb*2, together with detailed morphological examinations, confirmed that these collections belong to *Perennicordyceps*. Integrating molecular phylogenetic and morphological evidence, two previously undescribed species, *Pe. liupanshuiensis* and *Pe. tielingensis*, as well as a new host record for *Pe. prolifica*, were identified. These findings further expand the known diversity and host associations of *Perennicordyceps* in China.

## Materials and methods

### Sample collection, macro- and micromorphological examination

Five fresh specimens of *Perennicordyceps* were collected from insect hosts in Guizhou, Liaoning, and Yunnan Provinces, China. During field sampling, relevant metadata, including geographic coordinates and collection sites, were recorded following standard procedures (Rathnayaka et al. 2025). Specimens were transported to the laboratory in sterile plastic containers for further examination. In the laboratory, stromata were sectioned and examined under stereomicroscopes (Nikon SMZ 745 and SMZ 800N, Tokyo, Japan) to observe macroscopic characteristics. Micromorphological features, including perithecia, asci, ascospores, synnemata, conidiophores, phialides, and conidia, were observed and photographed using a Nikon DS-Ri2 digital camera attached to a Nikon ECLIPSE compound microscope, following previously described methods (Senanayake et al. 2020).

### Isolation and material deposition

Pure cultures were obtained by isolating mycelium from within the host tissue and transferring it onto potato dextrose agar (**PDA**) using a flame-sterilized needle under aseptic conditions. The cultures were incubated at 25 °C in the dark. The resulting strains were deposited in the Guizhou Culture Collection (**GZCC**), China, and dried specimens were deposited in the Herbarium of Cryptogams, Kunming Institute of Botany, Chinese Academy of Sciences (**HKAS**). Morphological measurements were performed using Tarosoft (R) v.0.9.7 Image Framework. Photographs were taken and processed using Adobe Photoshop CC 2022 (Adobe Systems, USA). Index Fungorum numbers were assigned to the newly described species following the guidelines of Index Fungorum.

### DNA extraction, PCR amplification, and sequencing

Genomic DNA was extracted from both dried specimens and cultures using the E.Z.N.A.® Plant & Fungal DNA Kit (Omega Bio-Tek, USA) following the manufacturer’s instructions. The extracted DNA was stored at −20 °C until use. Six gene regions, including the internal transcribed spacer (ITS), large subunit rDNA (nrLSU), small subunit rDNA (nrSSU), translation elongation factor 1-alpha (*tef*-1α), the largest subunit of RNA polymerase II (*rpb*1), and the second largest subunit of RNA polymerase II (*rpb*2), were amplified and sequenced using the primers listed in Table 1. PCR amplifications were carried out in a total volume of 25 μL, consisting of 2 μL of DNA template, 8.5 μL of nuclease-free water, 1 μL of each primer (10 μM), and 12.5 μL of 2× BenchTop™ Taq Master Mix (Biomiga, USA). The amplification protocol included an initial denaturation at 98 °C for 2 min, followed by 40 cycles of 98 °C for 10 s, 55 °C for 1 min, and 72 °C for 30 s, with a final extension at 72 °C for 2 min. PCR products were checked by electrophoresis on 1% (w/v) agarose gels prepared in 1× TAE buffer, stained with 4S Green Plus Nucleic Acid Stain (TSINGKE Biotech, China), and visualized under UV light. Sequencing was carried out by Tsingke Biotechnology (Chongqing, China). The newly generated sequences were deposited in GenBank, and accession numbers are provided in Table 2.

**Table 1.** Sequences of primers used in this study.

**Locus**	**Primers**	**Primer sequence (5'–3')**	**References**
ITS	ITS4	TCCTCCGCTTATTGATATGC	White et al. 1990
	ITS5	GGAAGTAAAAGTCGTAACAAGG	
nrSSU	NS1	GTAGTCATATGCTTGTCTC	White et al. 1990
	NS4	CTTCCGTCAATTCCTTTAAG	
nrLSU	LROR	ACCCGCTGAACTTAAGC	Vilgalys and Hester 1990
	LR5	TCCTGAGGGAAACTTCG	
*tef*-1α	EF1-983F	GCYCCYGGHCAYCGTGAYTTYAT	Carbone and Kohn 1999; Rehner and Buckley 2005
	EF1-2218R	ATGACACCRACRGCRACRGTYTG	
*rpb*1	CRPB1A	CAYCCWGGYTTYATCAAGAA	Castlebury et al. 2004
	RPB1Cr	CCNGCDATNTCRTTRTCCATRTA	
*rpb*2	fRPB2-5f	GAYGAYMGWGATCAYTTYGG	Castlebury et al. 2004
	fRPB2-7cR	CCCATRGCTTGYTTRCCCAT	

**Table 2.** Names, strain numbers, references, and corresponding GenBank accession numbers of the taxa used in the phylogenetic analyses of this study.

**Taxa names**	**Specimen/ strain number**	**GenBank accession numbers**						References
		**nrLSU**	**ITS**	**nrSSU**	***tef*-1α**	***rpb*1**	***rpb*2**	
*Cordyceps pleuricapitata*	NBRC 100745	JN941391	JN943304	JN941750	KF049679	JN992484	KF049667	Kepler et al. 2013
*Cordyceps pleuricapitata*	NBRC 100746	JN941392	JN943306	JN941749	KF049680	JN992483	KF049668	Kepler et al. 2013
*Dingleyomyces lloydii*	PDD 121254	OR602640	OR602634	OR647563	OR588853	OR588860	OR588858	Johnston and Park 2023
*Dingleyomyces yunnanensis*	HKAS 149970	PX688518	—	PX692988	PX694739	PX694719	—	Zhang et al. 2026a
*Paradingleyomyces lepidopterorum*	HKAS 131926	OR828238	OR878363	—	—	OR829674	OR880683	Wang et al. 2024
*Paradingleyomyces lepidopterorum*	HKAS 131927	OR828239	OR878364	—	OR880679	OR829675	—	Wang et al. 2024
*Perennicordyceps cuboidea*	NBRC 100941	JN941416	JN943329	JN941725	—	JN992459	—	Schoch et al. 2012
*Perennicordyceps cuboidea*	CEM 1514	KF049628	—	KF049609	KF049683	—	—	Kepler et al. 2013
*Perennicordyceps elaphomyceticola*	MFLU 21-0262	OQ172032	OQ172064	OQ172101	OQ459718	OQ459747	OQ459792	Xiao et al. 2023
*Perennicordyceps elaphomyceticola*	NTUCC 17-021	MK840812	MK840823	—	MK839229	MK839220	MK839211	Yang et al. 2020
***Perennicordyceps liupanshuiensis***	**HKAS 132267**	**PZ394736**	**PZ394729**	**PZ394743**	**PZ437645**	**PZ437635**	**PZ437639**	**This study**
***Perennicordyceps liupanshuiensis***	**HKAS 145891**	**PZ394737**	**PZ394730**	**PZ394744**	**PZ437646**	**PZ437636**	**PZ437640**	**This study**
*Perennicordyceps lutea*	KUMCC 3004	OQ474910	—	—	—	—	—	Xiao et al. 2023
*Perennicordyceps paracuboidea*	NBRC 101742	JN941431	JN943338	JN941710	KF049685	JN992444	KF049669	Schoch et al. 2012
*Perennicordyceps prolifica*	NBRC 103838	JN941434	JN943339	JN941707	—	JN992441	—	Schoch et al. 2012
*Perennicordyceps prolifica*	NBRC 101750	JN941433	JN943340	JN941708	—	JN992442	—	Schoch et al. 2012
***Perennicordyceps prolifica***	**HKAS 132166**	**PZ394738**	**PZ394731**	**PZ394745**	**PZ437647**	**PZ437637**	—	**This study**
*Perennicordyceps ryogamiensis*	NBRC 101751	JN941438	JN943343	JN941703	KF049688	JN992437	—	Schoch et al. 2012
*Perennicordyceps szemaoensis*	HKAS 36262	PV222164	PV222183	—	PV247101	PV247111	PV247106	Tang et al. 2025
*Perennicordyceps zongqii*	DY05421	PQ211282	PQ211278	—	PQ223679	—	PQ223677	Chen et al. 2024
***Perennicordyceps tielingensis***	**HKAS 132171**	**PZ394739**	**PZ394732**	**PZ394746**	**PZ437648**	**PZ437638**	**PZ437641**	**This study**
***Perennicordyceps tielingensis***	**GZCC 24-0254**	**OR727517**	**OR727503**	**OR727530**	**OR736002**	**OR736015**	**OR736026**	**This study**
*Perennicordyceps zongqii*	DY05422	PQ211283	PQ211279	—	PQ223680	—	PQ223678	Chen et al. 2024
*Pleurocordyceps agarica*	YHHPA1305	—	KP276651	KP276655	KP276659	KP276663	KP276667	Wang et al. 2015a
*Pleurocordyceps aurantiaca*	MFLUCC 17-2113	MG136910	MG136916	MG136904	MG136875	MG136866	MG136870	Xiao et al. 2018
*Pleurocordyceps clavisynnema*	GZCC 22-2042	OQ968797	OQ968789	OQ968805	OQ982008	OQ981998	OQ982004	Xiao et al. 2024
*Pleurocordyceps marginaliradians*	MFLU 17-2276	MG136915	MG136921	MG136909	MG136879	—	MG271930	Xiao et al. 2018
*Pleurocordyceps multisynnema*	GZCC 22-2041T	OQ968801	OQ968793	OQ968803	OQ982010	OQ981997	OQ982003	Xiao et al. 2024
*Pleurocordyceps nipponicus*	BCC 2325	KF049640	KF049665	KF049622	KF049696	KF049655	KF049677	Kepler et al. 2013
*Pleurocordyceps nutansis*	MFLU 21-0275	OQ172048	OQ172073	OQ172119	OQ459739	OQ459765	OQ459811	Xiao et al. 2023
*Pleurocordyceps sanduensis*	GZCC 22-2044	OQ968799	OQ968787	OQ968806	OQ982006	OQ981995	OQ982001	Xiao et al. 2024
*Pleurocordyceps sinensis*	CN 80-2	HQ832886	HQ832884	HQ832887	HQ832890	HQ832888	HQ832889	Wang et al. 2012
*Pleurocordyceps tengchongensis*	HKAS 149971	PX688520	PX692975	PX692990	PX694741	PX694721	PX694729	Zhang et al. 2026a
*Pleurocordyceps vitellina*	KUMCC 3006	OQ172061	OQ172089	—	OQ459729	OQ459757	OQ459803	Xiao et al. 2023
*Pleurocordyceps yunnanensis*	YHCPY 1005	—	KF977848	—	KF977850	KF977852	KF977854	Wang et al. 2015b
*Polycephalomyces albiramus*	GACP 21-XS08	OQ172037	OQ172092	OQ172115	OQ459735	OQ459761	OQ459807	Xiao et al. 2023
*Polycephalomyces bannaensis*	HKAS 149954	PX688514	PX688512	PX692982	PX694734	PX694716	—	Zhang et al. 2026a
*Polycephalomyces chiangraiensis*	MFLU 26-0001	PX688524	PX692979	PX692994	PX694745	PX694725	—	Zhang et al. 2026a
*Polycephalomyces formosus*	NBRC 100686	MN586839	MN586830	MN586821	MN598054	MN598045	MN598061	Wang et al. 2020
*Polycephalomyces myrmecophilus*	YFCC 09289444	PP410606	PP410603	PP410609	PP581796	PP697741	PP581813	Liu et al. 2024
*Polycephalomyces tengchongensis*	HKAS 131923	OR828240	OR878365	PP129612	—	OR829676	OR880685	Wang et al. 2024
*Torrubiellomyces zombiae*	NY04434801	ON493602	—	ON493543	ON513396	ON513398	ON513402	Araújo et al. 2022
*Torrubiellomyces zombiae*	FieldB	ON493603	—	ON493544	ON513395	—	—	Araújo et al. 2022
*Ophiocordyceps sinensis*	EFCC 7287	EF468827	JN049854	EF468971	EF468767	EF468874	EF468924	Sung et al. 2007a
*Ophiocordyceps stylophora*	OSC 111000	DQ518766	JN049828	DQ522552	DQ522337	DQ522382	DQ522433	Spatafora et al. 2007

Note: The symbol “—” indicates that the sequence is not available; newly generated sequences in this study are shown in bold.

### Phylogenetic analyses

Newly obtained sequences were assembled with SeqMan v. 11.1.0 (DNASTAR, Inc., Madison, WI, USA). Candidate taxa for phylogenetic reconstruction, including reference and closely related species, were identified through BLAST searches in NCBI GenBank and by consulting the relevant literature (Schoch et al. 2012; Kepler et al. 2013; Johnston and Park 2023; Xiao et al. 2023; Chen et al. 2024; Tang et al. 2025; Zhang et al. 2026a) (Table 2). Both newly generated sequences and those retrieved from public databases were incorporated into subsequent analyses.

Each locus was aligned independently using MAFFT v. 7 under the “auto” strategy (Katoh and Standley 2013). Poorly aligned regions were removed with TrimAl, employing the “gappyout” algorithm (Capella-Gutiérrez et al. 2009). The optimal substitution model for each partition was determined according to the Bayesian information criterion (BIC) using ModelFinder (Kalyaanamoorthy et al. 2017). Individual alignments were concatenated into a combined dataset and partitioned prior to phylogenetic reconstruction.

Maximum likelihood (ML) analyses were performed using RAxML-HPC2 (Stamatakis 2014) via the CIPRES Science Gateway v. 3.3 (Miller et al. 2010), applying default parameters and 1,000 bootstrap replicates. Bayesian inference (BI) was conducted in MrBayes v. 3.1.2 (Ronquist et al. 2012) under the GTR+I+G model. Six Markov chains were run for 5,000,000 generations, with sampling every 100 generations. The analysis was considered to have converged when the average standard deviation of split frequencies dropped below 0.01. Convergence diagnostics were evaluated in Tracer v. 1.6 (Rambaut et al. 2013). The initial 25% of trees were discarded as burn-in, and the remaining trees were used to calculate posterior probabilities (PP). *Ophiocordyceps sinensis* (EFCC 7287) and *O. stylophora* (OSC 111000) were designated as outgroup taxa. Node support was interpreted as significant when ML bootstrap values were ≥ 75% and BI posterior probabilities were ≥ 0.90. The final tree was visualized using FigTree v. 1.4.0 (Rambaut 2012).

### Phylogenetic analysis results

**Figure 1.**
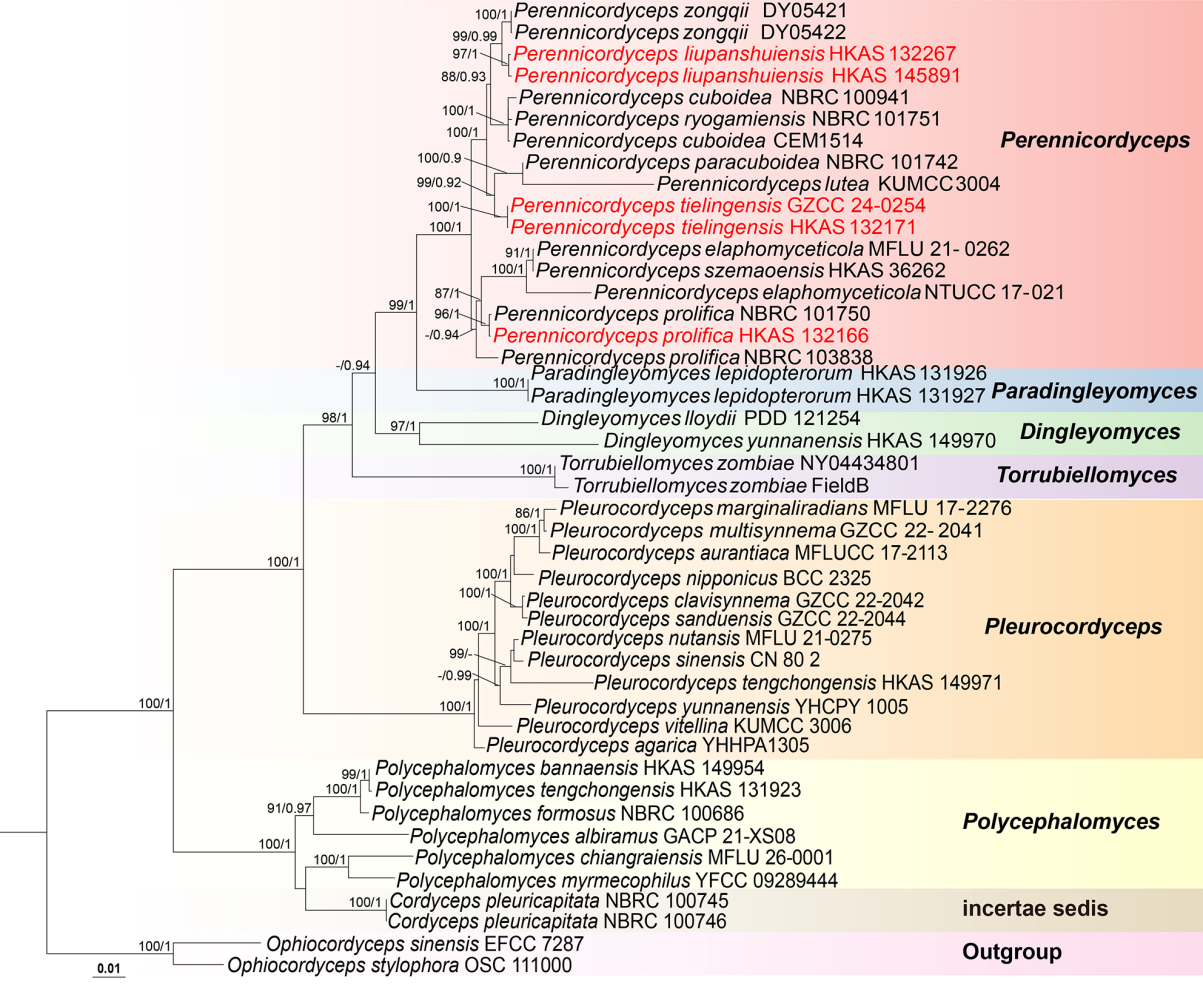
A phylogenetic tree was inferred based on ML analysis using RAxML, incorporating combined sequence data from six nuclear loci, including nrLSU, ITS, nrSSU, *tef*-1α, *rpb*1, and *rpb*2. *Ophiocordyceps sinensis* (EFCC 7287) and *O. stylophora* (OSC 111000) were designated as outgroup taxa. Node support values are indicated on the phylogram, with ML bootstrap values ≥ 75% and Bayesian posterior probabilities (PP) ≥ 0.90 considered significant. Newly generated sequences are highlighted in red.

A combined dataset comprising nrLSU, ITS, nrSSU, *tef*-1α, *rpb*1, and *rpb*2 sequences was assembled, including 45 strains representing 36 taxa. The final alignment consisted of 5,021 characters, partitioned as follows: nrLSU (1–840 bp), ITS (841–1382 bp), nrSSU (1383–2397 bp), *tef*-1α (2398–3303 bp), *rpb*1 (3304–3984 bp), and *rpb*2 (3985–5021 bp). Phylogenetic analyses based on ML and BI produced congruent topologies. The best-scoring ML tree inferred by RAxML had a final log-likelihood of −26808.351968 (Fig. 1). Estimated base frequencies were A = 0.240520, C = 0.268247, G = 0.276998, and T = 0.214234, and substitution rates were AC = 1.345496, AG = 3.813126, AT = 1.024925, CG = 1.139246, CT = 7.463566, and GT = 1.000000. The gamma distribution shape parameter (α) was estimated at 0.596997. Overall, the phylogenetic reconstructions recovered consistent relationships among taxa. Based on these results, two new species, *Perennicordyceps liupanshuiensis* and *Pe. tielingensis*, are proposed, and a new host record is reported for *Pe. prolifica*.

## Taxonomy

### Perennicordyceps liupanshuiensis

Taxon classificationFungiHypocrealesOphiocordycipitaceae

Y. Yang, B.X. Zhang & Y. P. Xiao sp. nov.

AF75319D-29D6-53D7-8E76-A1C2045B9396

Index Fungorum: IF905474

Figs 2, 3

#### Etymology.

The epithet “*liupanshuiensis*” refers to the type location “Liupanshui City, China.”

#### Holotype.

China • Guizhou Province, Liupanshui City, Shuicheng County, occurs on the larvae of *Coleoptera*, on leaf litter, 26°27'43.18"N, 104°48'37.98"E, 2377 m elev., 31 July 2022, Jing-Yi Zhang, SC194 (HKAS 132267, Herbarium of Cryptogams, Kunming Institute of Botany, Chinese Academy of Sciences).

**Figure 2.**
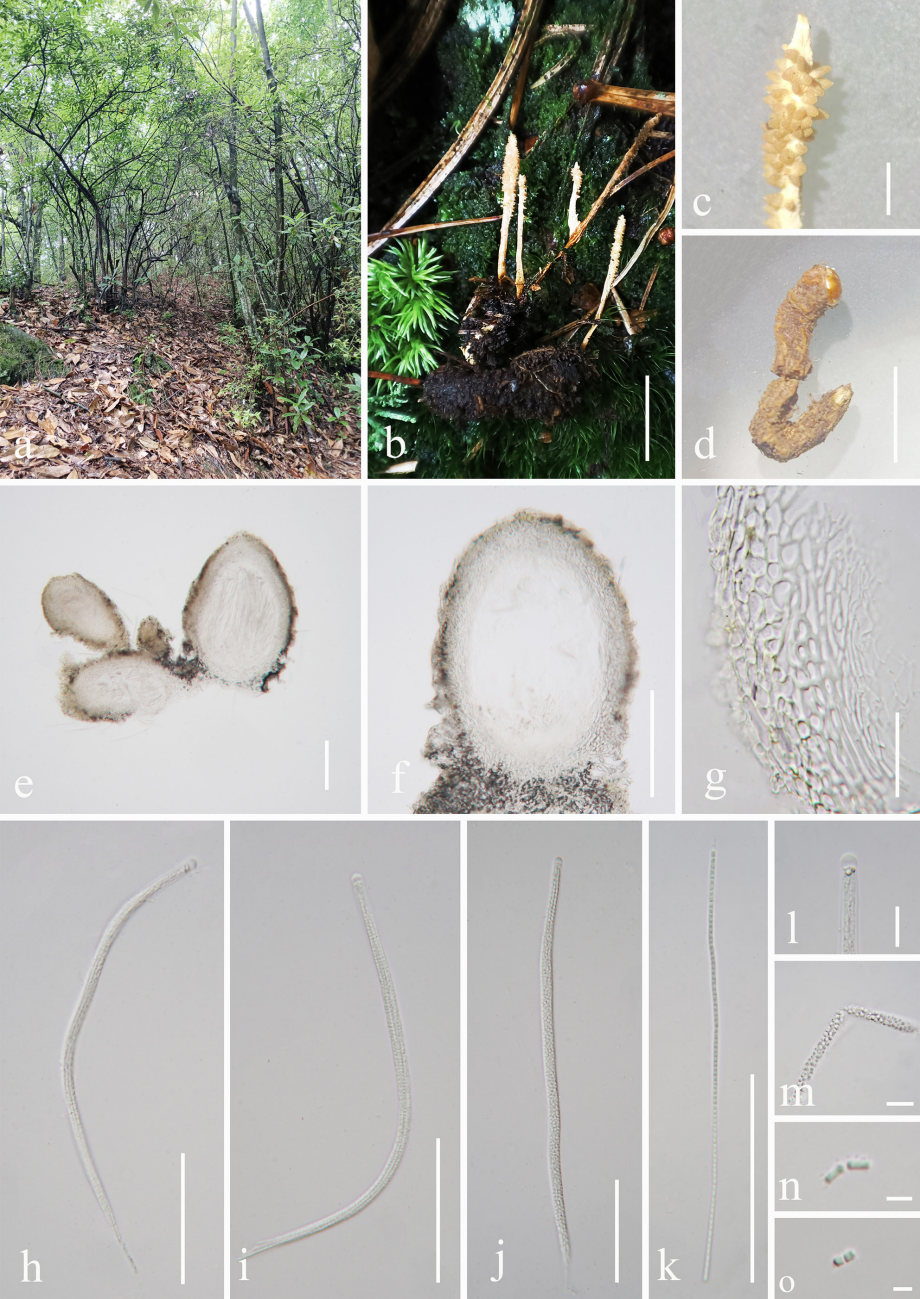
*Perennicordyceps liupanshuiensis* (HKAS 132267, holotype). **a**, **b**. Habitat; **c**. Fertile head of ascostroma; **d**. Overview of host; **e**, **f**. Perithecia; **g**. Peridium; **h**–**j**. Asci; **k**. Part of ascospore; **l**. Apical cap of asci; **m**–**o**. Secondary ascospores. Scale bars: 1 cm (**b, d**); 0.2 cm (**c**); 100 µm (**e, f**); 20 µm (**g**); 50 µm (**h–k**); 10 µm (**l, m**); 3 µm (**n, o**).

#### Description.

Parasitic on the larvae of *Coleoptera*. ***Host*** 2.1–3.3 × 0.2–0.4 cm, reddish-brown, without hyphae on the surface. **Sexual morph: *Stromata*** 2.4–3.5 × 0.18–0.32 cm diam., mostly single, cylindrical, unbranched, emerging from the head of the larva body, yellowish-white. ***Fertile head*** 4.5 × 6 mm, subglobose, pale yellow when fresh, pale yellow-brown when dry, distinct from the stipe. ***Stipe*** 1.5–2.3 × 0.18–0.21 cm, pale yellow, straight, unbranched, glossy, cylindrical, inside not hollow. ***Perithecia*** 255–356 × 155–231 μm (x̄ = 305.5 × 193 µm, *n* = 30), superficial, ampulliform, ostiolate, thick-walled. ***Asci*** 170–208 × 4.6–5.7 μm (x̄ = 189 × 5.15 µm, *n* = 30), hyaline, filiform, with a thin apex. ***Ascospores*** filiform, equal to the asci in length, when mature, breaking into numerous secondary ascospores. ***Secondary ascospores*** 1.3–3.1 × 1.1–1.3 µm (x̄ = 2.2 × 1.2, *n* = 40), short cylindrical, one-celled, straight, hyaline, smooth. **Asexual morph: *Synnemata*** 3.5–8.5 × 0.5–1.2 mm, numerous, slender, erect, white, arising from the host and surrounding substrate, scattered to gregarious, not distinctly swollen at the apex. ***Hyphae*** forming a superficial layer on the host surface, from which conidiogenous structures arise. ***Conidiophores*** reduced, not clearly differentiated. ***Phialides*** 8.7–19.5 × 1.8–2.7 µm (x̄ = 14.4 × 2.25, *n* = 40), solitary, arising directly from hyphae, short, slender, slightly tapering. ***Conidia*** 3.3–4.5 × 1.5–2.1 µm (x̄ = 3.9 × 1.8, *n* = 40), hyaline, smooth-walled, one-celled, ellipsoid to ovoid.

**Figure 3.**
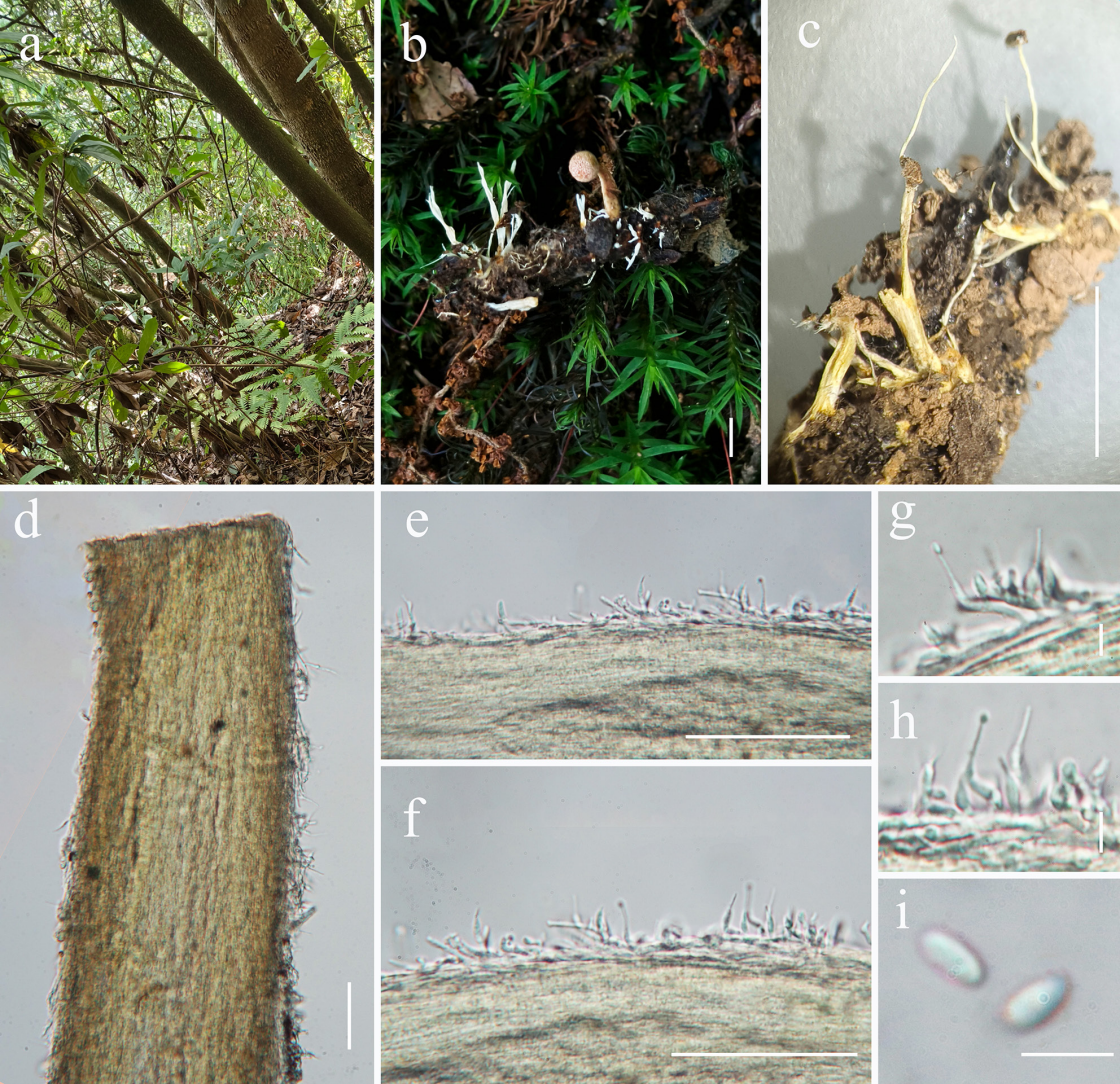
*Perennicordyceps liupanshuiensis* (HKAS 145891, paratype). **a**. Habitat; **b**. Overview of host; **c**. *Perennicordyceps liupanshuiensis* on host; **d**. Mycelium under microscope; **e**–**h**. Conidiophores and phialides; **i**. Conidia. Scale bars: 0.5 cm (**b, c**); 50 μm (**d–f**); 5 μm (**g–i**).

#### Culture characters.

Not observed. Repeated isolation attempts were made from both sexual and asexual specimens; however, no stable culture was obtained.

#### Other material examined.

China • Yunnan Province, Honghe Hani and Yi Autonomous Prefecture, Honghe County, parasitic on a larva of Coleoptera, buried in the soil, 23°16'15.60"N, 102°17'27.60"E, 1963 m elev., 18 July 2024, Yu Yang, YY24345-a (HKAS 145891, paratype).

#### Notes.

Multilocus phylogenetic analyses place *Perennicordyceps liupanshuiensis* in a well-supported clade within *Perennicordyceps*, closely related to *Pe. zongqii* (Fig. 1). Since *Pe. zongqii* is known only from its asexual morph, comparison between the two species is restricted to asexual characteristics. *Perennicordyceps liupanshuiensis* differs from *Pe. zongqii* in having shorter and simpler phialides (8.7–19.5 × 1.8–2.7 μm vs. 29.3–31.1 × 1.5–2.4 μm), which arise directly from hyphae, whereas *Pe. zongqii* produces well-developed conidiophores bearing multiple phialides (Chen et al. 2024). Ecologically, *Pe. liupanshuiensis* occurs on larvae of *Coleoptera*, whereas *Pe. zongqii* has been reported from *Lepidoptera* larvae (Chen et al. 2024). Pairwise sequence comparisons between *Pe. liupanshuiensis* and its closest relatives show differences of 1.5% (10/655 bp) in ITS, 1.7% (15/874 bp) in *tef*-1α, and 2.1% (20/950 bp) in *rpb*2 (Chen et al. 2024). Therefore, *Perennicordyceps liupanshuiensis* is recognized as a distinct species based on morphological and phylogenetic evidence.

### Perennicordyceps prolifica

Taxon classificationFungiHypocrealesOphiocordycipitaceae

(Kobayasi) Matočec & I. Kušan, in Matočec, Kušan & Ozimec, Ascomycete.org 6(5): 129 (2014)

5AED81C3-B19D-54EB-87D3-5332F18F10C7

Index Fungorum: IF810776

Fig. 4

#### Description.

Parasitic on a lepidopteran cocoon, on leaf litter. ***Host*** 4–5 cm long, elongate, pale brown, smooth, attached to substrate. **Sexual morph**: Undetermined. **Asexual morph: *Synnemata*** 5–6 cm long, solitary, arising from the anterior part of the host, cylindrical, straight, white, with a slightly tapering apex. ***Fertile region*** not differentiated from the stipe, the surface appearing slightly roughened. ***Conidiophores*** hyaline, sparse, arising from the surface, simple. ***Phialides*** 18.5–29.8 × 0.8–1.8 μm (x̄ = 16.5 × 2.3 µm, *n* = 40), hirsutella-like, hyaline, slender, tapering gradually towards the apex. ***Conidia*** 2.2–3.6 × 1.2–2.3 μm (x̄ = 16.5 × 2.3 µm, *n* = 40), one-celled, hyaline, smooth-walled, ellipsoidal.

**Figure 4.**
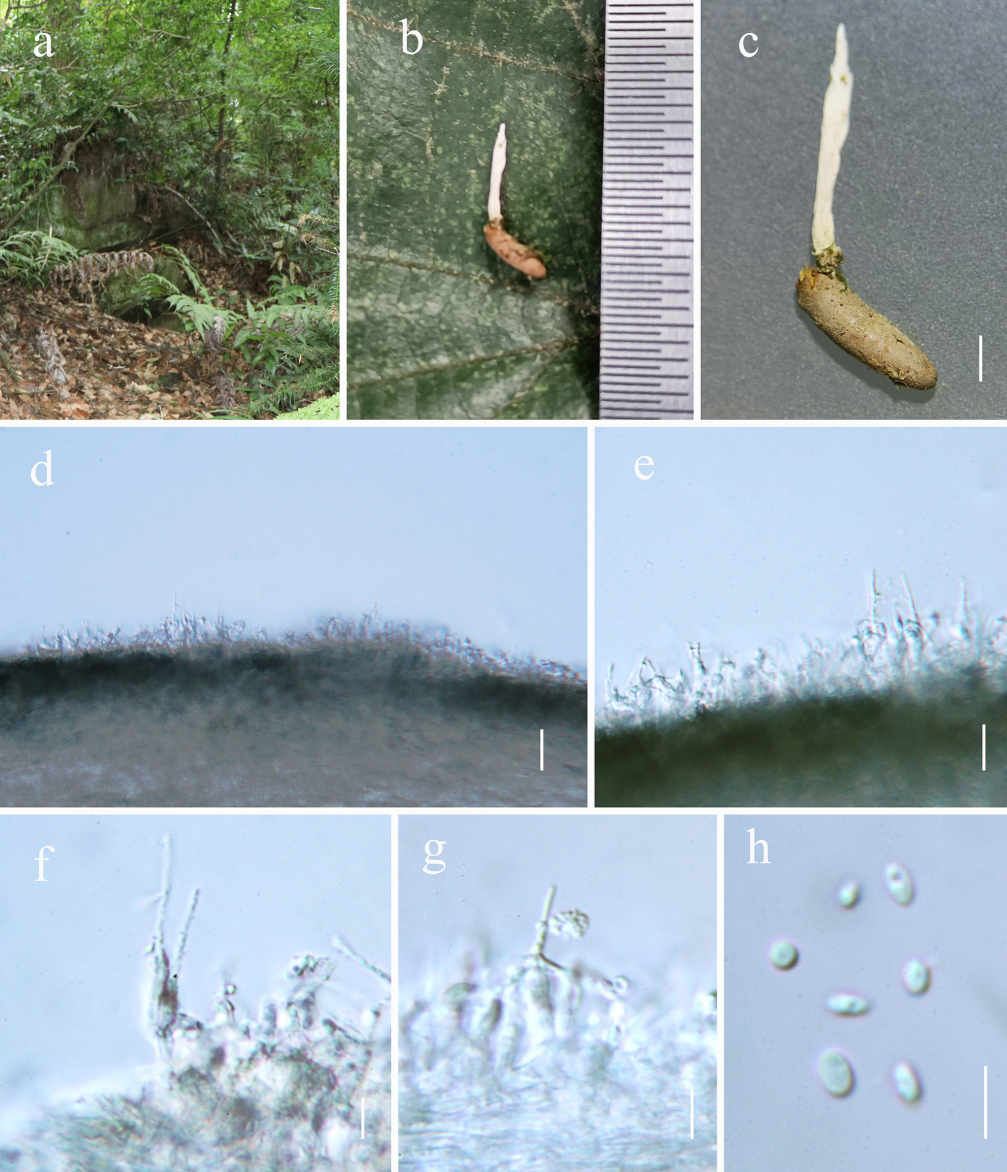
*Perennicordyceps prolifica* (HKAS 132166, new record). **a**. Habitat; **b**–**c**. Overview of host; **d**–**g**. Conidiophores and phialides; **h**. Conidia. Scale bars: 0.1 cm (**c**); 20 μm (**d**); 10 μm (**e**); 5 μm (**f**–**h**).

#### Material examined.

China • Guizhou Province, Zunyi City, Xishui County, parasitic on a cocoon of *Lepidoptera*, buried in the soil, 28°30'1.31"N, 106°23'44.76"E, 967 m elev., 10 April 2023, Yu Yang, XS2304 (HKAS 132166).

#### Culture characters.

Not observed. Repeated isolation attempts were made from specimens; however, no stable culture was obtained.

#### Notes.

Phylogenetic analyses place the present strain (HKAS 132166) within *Perennicordyceps prolifica*, where it forms a strongly supported lineage with strain NBRC 101750 (96% MLBP, 1.00 PP; Fig. 1). Morphologically, the specimen agrees well with the description of *Pe. prolifica*, particularly in producing slender, cylindrical synnemata and hirsutella-like phialides, as well as comparable conidial size and shape (Matočec et al. 2014). *Perennicordyceps prolifica* was originally described from underground-dwelling nymphs of *Cicadidae (Hemiptera)* (Kobayasi and Shimizu 1963; Matočec et al. 2014). In contrast, the present specimen was collected from a lepidopteran cocoon, representing a new host record for the species. Furthermore, *Pe. prolifica* has previously been reported from Japan (Kobayasi and Shimizu 1963; Matočec et al. 2014), whereas the present collection from China represents a new geographic record. These results suggest that *Pe. prolifica* has a broader host range and wider distribution than previously recognized.

### Perennicordyceps tielingensis

Taxon classificationFungiHypocrealesOphiocordycipitaceae

Y. Yang, B.X. Zhang & Y. P. Xiao sp. nov.

82BFB6D1-C3B5-59E6-8F3D-34F539FAEE35

Index Fungorum: IF905475

Fig. 5

#### Etymology.

The epithet “*tielingensis*” refers to the type location “Tieling City, China.”

#### Holotype.

China • Liaoning Province, Tieling City, Xifeng County, occurs on the stipe of *Ophiocordyceps* sp., on leaf litter, 42°39'10.80"N, 124°27'7.20"E, 385 m elev., 22 July 2023, Yuan-Pin Xiao, TL24 (HKAS 132171, Herbarium of Cryptogams, Kunming Institute of Botany, Chinese Academy of Sciences), ex-type living culture TL24B (= GZCC 24-0254, Guizhou Culture Collection, China).

#### Description.

Parasitic on *Ophicordyceps* sp. (*Ophiocordycipitaceae*, *Hypocreales*), collected from the underside of leaves. **Sexual morph**: Undetermined. **Asexual morph**: Hyphomycetous. ***Synnemata*** 5–17 mm long, 0.5–1.3 mm wide on the stipe of *Ophiocordyceps* sp., solitary or several, white to pale yellow, cylindrical without an enlarged globose fertile head on the top, without secondary synnemata. ***Conidiophores*** 20–35 × 2.2–3.6 μm (x̄ = 27.5 × 2.9 µm, *n* = 50), hyaline, produced on aerial mycelium, usually branched into 2–5 phialides. ***Phialides*** 5–16 × 1.2–2.5 μm (x̄ = 10.5 × 1.9 µm, *n* = 50), only one type observed, narrow slender lanceolate, usually 1–2 phialides in one, 2 branched on one metula hyaline. ***Conidia*** 2.4–3.6 × 1.5–2.5 μm (x̄ = 2.4, *n* = 50), ellipsoid, hyaline, one-celled, smooth-walled.

**Figure 5.**
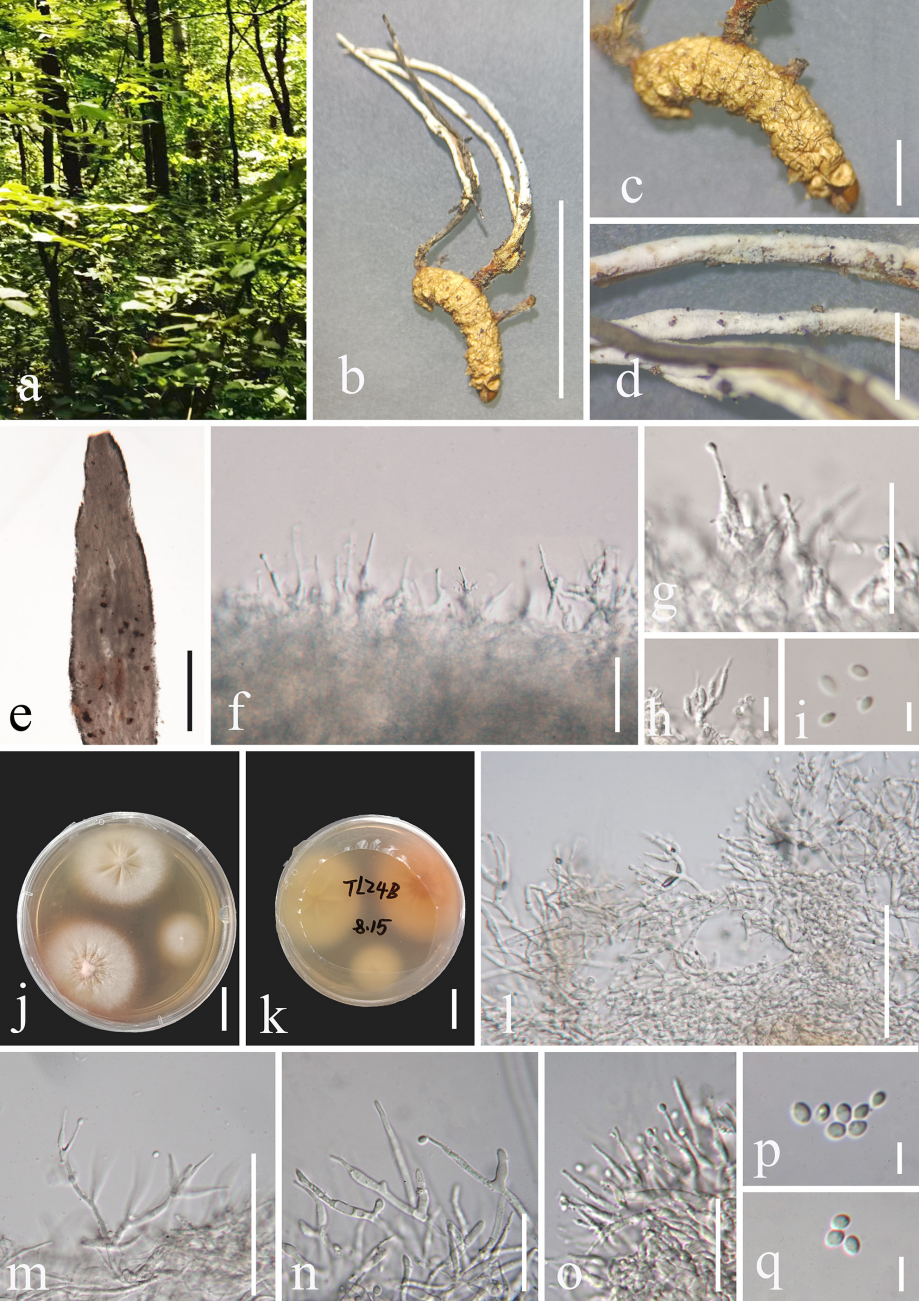
*Perennicordyceps tielingensis* (HKAS 132171, holotype). **a**. Habitat; **b**. Overview of host; **c**. Host of *Ophiocordyceps* sp; **d**. Synnemata; **e**. Synnema under microscope; **f**, **g**. Conidiophores; **h**. Phialides; **i** Conidia; **j**, **k**. Colonies on PDA (obverse and reverse); **l**–**o**. Conidiophores and phialides from culture; **p**, **q**. Conidia from culture. Scale bars: 1 cm (**b**); 0.2 cm (**c, d**); 20 µm (**e, f**); 10 µm (**g**); 5 µm (**h**); 1 cm (**j, k**); 50 µm (**l, m**); 20 µm (**n, o**); 5 µm (**p, q**).

#### Culture characters.

Colonies derived from tissue isolation. Colonies on PDA slowly growing, reached to 2 cm diam in 35 days at 25 °C, fluffy with lower mycelial density, milky white to pale yellow, same color on the reverse, no synnemata observed. ***Conidiophores*** 24–55 µm long (x̄ = 39.5 µm, *n* = 50), 1–3 phialides in one. ***Phialides*** 8–25 × 1.6–3 μm (x̄ = 16.5 × 2.3 µm, *n* = 50) hyaline, smooth, hirsutella-like, narrow cylindrical at the low base part, tapering into a long neck. ***Conidia*** 2.1–3.2 × 1.5–2.2 µm (x̄ = 2.6 × 1.9 µm, *n* = 50), one-celled, hyaline, ellipsoid.

#### Notes.

*Perennicordyceps tielingensis* clustered with *Pe. lutea* and *Pe. paracuboidea* in the phylogenetic tree with 99% MLBP and 0.92 PP support (Fig. 1). Pairwise sequence comparisons revealed differences of 2.4% (13/543 bp) in ITS, 4.2% (35/841 bp) in nrSSU, 3.6% (31/866 bp) in *tef*-1α, 1.7% (12/718 bp) in *rpb*1, and 2.6% (26/986 bp) in *rpb*2 between *Pe. tielingensis* and *Pe. paracuboidea* (Schoch et al. 2012). For *Pe. lutea*, only nrLSU sequence data are available; therefore, sequence comparisons were limited to this locus, showing 2.1% (18/840 bp) differences between *Pe. tielingensis* and *Pe. lutea* (Xiao et al. 2023). The host of *Pe. tielingensis* is the stipe of *Ophiocordyceps* sp., whereas *Pe. paracuboidea* has been reported from *Coleoptera* hosts, and *Pe. lutea* is associated with *Ophiocordyceps sinensis* (Ban et al. 2009; Xiao et al. 2023). Compared to *Pe. lutea*, *Pe. tielingensis* produces shorter phialides (5–16 μm vs. 14.8–64.9 μm) and smaller conidia (2.4–3.6 × 1.5–2.5 μm vs. 2.3–4.5 × 1.8–3.4 μm) (Xiao et al. 2023). Compared to *Perennicordyceps paracuboidea*, *Pe. tielingensis* produces shorter phialides (5–16 μm vs. 10.9–21.5 μm) and larger conidia (2.4–3.6 × 1.5–2.5 μm vs. 1.3–1.9 × 1–1.9 μm) (Ban et al. 2009). Therefore, both morphological and phylogenetic evidence support *Perennicordyceps tielingensis* as a distinct new species.

## Discussion

The present study expands the known diversity and host associations of *Perennicordyceps* by introducing two novel taxa and reporting a new host record. Phylogenetic analyses based on combined multigene data, together with morphological comparisons, strongly support the recognition of *Pe. liupanshuiensis* and *Pe. tielingensis* as distinct taxa within *Perennicordyceps*. Morphological differences in asexual structures, particularly phialide morphology, conidiophore organization, and conidial dimensions, were consistent with phylogenetic separation among species. These results further highlight the taxonomic importance of asexual characteristics in clavicipitoid fungi, especially in taxa for which sexual morphs are absent or rarely observed (Taylor et al. 1999; Ban et al. 2009; Maharachchikumbura et al. 2021).

Beyond their taxonomic significance, the discovery of *Pe. tielingensis* parasitizing the stipe of *Ophiocordyceps* provides additional evidence of ecological diversity within *Perennicordyceps*. Similar fungicolous and hyperparasitic associations have been reported in related members of *Polycephalomycetaceae*, including *Polycephalomyces jinghongensis* parasitizing *Ophiocordyceps* species and *Pleurocordyceps parvicapitata* associated with *Elaphomyces* sp. (Xiao et al. 2023; Liu et al. 2024). These observations suggest that parasitism on other fungi may represent a recurrent ecological strategy within *Polycephalomycetaceae* (Wang et al. 2012, 2015a, 2015b; Matočec et al. 2014; Xiao et al. 2023, 2024; Zhang et al. 2026a, 2026b). The new host record of *Pe. prolifica* from a lepidopteran cocoon expands its previously known association with cicada nymphs (*Hemiptera*) (Kobayasi and Shimizu 1963; Matočec et al. 2014). Comparable patterns of multiple host associations have also been documented in related taxa, indicating that some species of *Polycephalomycetaceae* may exhibit broader host ranges than previously recognized (Bischoff et al. 2003; Ban et al. 2009; Chen et al. 2024). These observations suggest that host associations within *Perennicordyceps* may be more diverse than previously recognized.

Previous studies have shown that species of clavicipitoid fungi can produce diverse bioactive secondary metabolites with antibacterial, antifungal, and other biological activities (Sangdee et al. 2015, 2016; Thammawat et al. 2017; Jia et al. 2025; Yu et al. 2025). However, studies focusing on *Perennicordyceps* remain limited. Considering the ecological diversity observed within the genus, including both entomopathogenic and fungicolous lifestyles, *Perennicordyceps* may represent a promising source of unexplored metabolites. Further studies integrating taxonomy, ecology, and natural product research will improve the understanding of the evolutionary and biological significance of this lineage.

## Supplementary Material

XML Treatment for Perennicordyceps liupanshuiensis

XML Treatment for Perennicordyceps prolifica

XML Treatment for Perennicordyceps tielingensis

## Additional information

### Artificial Intelligence (AI) use

The authors accept full responsibility for the content of the manuscript, including the disclosure of any use of AI.

No AI tools were used in the preparation of this manuscript.

### Author ORCIDs

Y. Yang https://orcid.org/0000-0001-8268-487X

J.-Y. Zhang https://orcid.org/0000-0003-0606-6169

K.D. Hyde https://orcid.org/0000-0002-2191-0762

Y.-Z. Lu https://orcid.org/0000-0002-1033-5782

Y.-P. Gong https://orcid.org/0000-0001-5794-6727

Y.-P. Xiao https://orcid.org/0000-0003-1730-3545

### Data availability

All of the data that support the findings of this study are available in the main text.
